# UAV reveals substantial but heterogeneous effects of herbivores on Arctic vegetation

**DOI:** 10.1038/s41598-021-98497-5

**Published:** 2021-09-30

**Authors:** Matthias B. Siewert, Johan Olofsson

**Affiliations:** Department of Ecology and Environmental Science, Umeå University, Umeå, Sweden

**Keywords:** Climate-change ecology, Ecosystem ecology, Climate-change ecology

## Abstract

Understanding how herbivores shape plant biomass and distribution is a core challenge in ecology. Yet, the lack of suitable remote sensing technology limits our knowledge of temporal and spatial impacts of mammal herbivores in the Earth system. The regular interannual density fluctuations of voles and lemmings are exceptional with their large reduction of plant biomass in Arctic landscapes during peak years (12–24%) as previously shown at large spatial scales using satellites. This provides evidence that herbivores are important drivers of observed global changes in vegetation productivity. Here, we use a novel approach with repeated unmanned aerial vehicle (UAV) flights, to map vegetation impact by rodents, indicating that many important aspects of vegetation dynamics otherwise hidden by the coarse resolution of satellite images, including plant–herbivore interactions, can be revealed using UAVs. We quantify areas impacted by rodents at four complex Arctic landscapes with very high spatial resolution UAV imagery to get a new perspective on how herbivores shape Arctic ecosystems. The area impacted by voles and lemmings is indeed substantial, larger at higher altitude tundra environments, varies between habitats depending on local snow cover and plant community composition, and is heterogeneous even within habitats at submeter scales. Coupling this with spectral reflectance of vegetation (NDVI), we can show that the impact on central ecosystem properties like GPP and biomass is stronger than currently accounted for in Arctic ecosystems. As an emerging technology, UAVs will allow us to better disentangle important information on how herbivores maintain spatial heterogeneity, function and diversity in natural ecosystems.

## Introduction

Ecologists have long aimed to acquire a better spatial understanding of the influence herbivores have on vegetation^1,2^. Herbivores are worldwide known to influence plant biomass, community structure and diversity^2–4^, but the impact of herbivores is not spread even across landscapes^5,6^. There are many drivers of heterogeneity in herbivore density, behaviour and finally impact on the vegetation. The most obvious drivers might be physical barriers such as steeps or water bodies making areas unavailable for herbivores^7^. An equally self-evident driver of spatial heterogeneity is food availability. Herbivores are known to select the landscape unit richest in resources, then the most productive communities within the landscape, down to the most palatable species within a feeding station. This creates a gradient of herbivory intensity across scales^8^. Access to other resources such as water^5^, minerals^9^ or burrows^10^ might also generate heterogeneity in herbivore distribution and impact on the vegetation. Predators are another factor generating heterogeneity in herbivore impact on the vegetation through trophic cascades^11^. The density of predators might determine the impact of herbivores on the vegetation via population^12,13^ or behaviour responses^1^.

To understand the importance of herbivory for ecosystem structure and function, we do not only need to understand the intensity of the interactions between herbivores and plants, but also their spatial heterogeneity^5^. Different plant communities respond in contrasting ways to the same level of herbivory^14^, and the same habitats may respond differently to patchy and homogenous grazing^5^. Therefore, different spatial configurations of herbivory will impact ecosystem heterogeneity at different spatial scales^5^. Heterogeneous landscapes are expected to have a higher species diversity^15^, higher stability^16^ and a higher ability to buffer effects of a warming climate^7^. A high spatial heterogeneity within the scale of our measurement might also cause underestimation of vital ecosystem properties such as plant biomass, GPP or soil organic carbon^17,18^.

**Figure 1:**
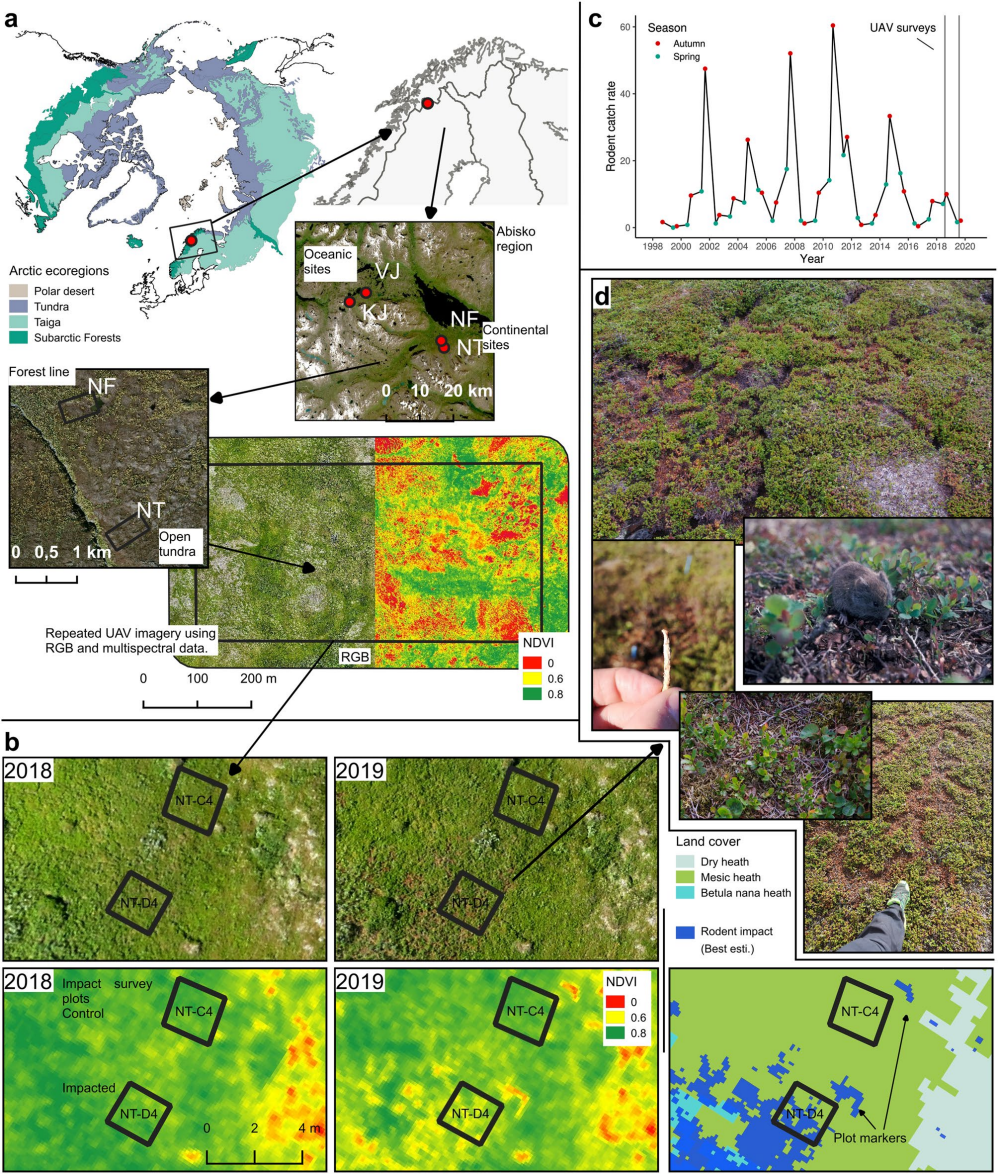
(**a**) Location of the study area in the Arctic. Ecoregions of the Tundra and Boreal Forest/Taiga biomes^30^ where vole and lemming cycles are common^22^. Study setup with four study areas paired for oceanic and continental climate, and low altitude forest line tundra and higher altitude open tundra. RGB and multispectral UAV imagery was recorded at all sites and the NDVI was derived as an index for vegetation productivity^6^. (**b**) UAV imagery for 2018 and 2019 of the same selected area with ground truth plots showing rodent impact in form of nests and runways that appear as brown areas in the RGB imagery (**b** top row) and as reduced NDVI (**b** bottom row left and middle) in previously homogeneous vegetation cover of mesic tundra. Mapped land cover and rodent impact for the same area (**b** bottom row right). (**c**) Combined regional vole and lemming catch rates for 1998–2020. Vertical line indicate time of UAV flights in summer 2018 and 2019. The time-series indicates a comparatively low rodent peak for 2018. See Supplementary Fig. S1 in the supplement for detailed time-series differentiating for voles, lemmings, oceanic and continental area. (**d**) Images show a grey sided vole harvesting vegetation and examples of the impact on vegetation and traces left by rodents including runways, clipped vegetation, woody remains with bite marks and feces droppings. Figure created in QGIS 3.18 (qgis.org), Made with Natural Earth (naturalearthdata.com), Regional imagery: European Environment Agency (EEA) and Lantmäteriet.

Compared to the comprehensive understanding of plant–herbivore interactions in general, the knowledge about the variation of these interactions in space is still rudimental. To a large part this is due to limited capability of remote sensing to assess the effect of herbivory^3^. This knowledge gap reflects the difficulty of studying and quantifying the heterogeneity at scales relevant to mammalian herbivores. The strong population fluctuation of many herbivores in northern ecosystems^19^ provides a unique opportunity to study the impact of herbivores at large spatial scales by comparing conditions before and after a population peak using remote sensing techniques. This approach has been used to study the impact of different types of herbivores ranging from reindeer population peaks^20^ to insect outbreaks^21^. One spectacular example of such fluctuations are vole and lemming cycles that are a common feature of Arctic ecosystems^22^, and drive synchronous interannual fluctuations in biomass of field-layer vegetation with reductions of (12–24%) during cycle peak years, which are visible as a reduced normalized difference vegetation index (NDVI) in satellite images in the following year^23^. The impact of the voles and lemmings on the vegetation is a heterogeneous mix of heavily impacted runways and patches around burrows^24^, where the total removal of vegetation results in strongly reduced NDVI, intermingling with virtually unaffected areas (Fig. 1). The coarse resolution of satellite images severely restricts the ability to study the spatial distribution of the herbivore impact, especially in Arctic landscapes which are highly heterogeneous at meter to sub-meter scales^17,25–27^. Unmanned aerial vehicles (UAVs) have emerged as a new technology in ecology^28^ and provide the opportunity to survey landscapes over large areas at high spatial resolution^17,29^. Low cost and temporal flexibility allow to use them for seasonal monitoring or targeted field campaigns ^17,28^ and they have proven to provide reliable truecolor and multispectral data even in challenging Arctic locations ^17,29^.

**Figure 2:**
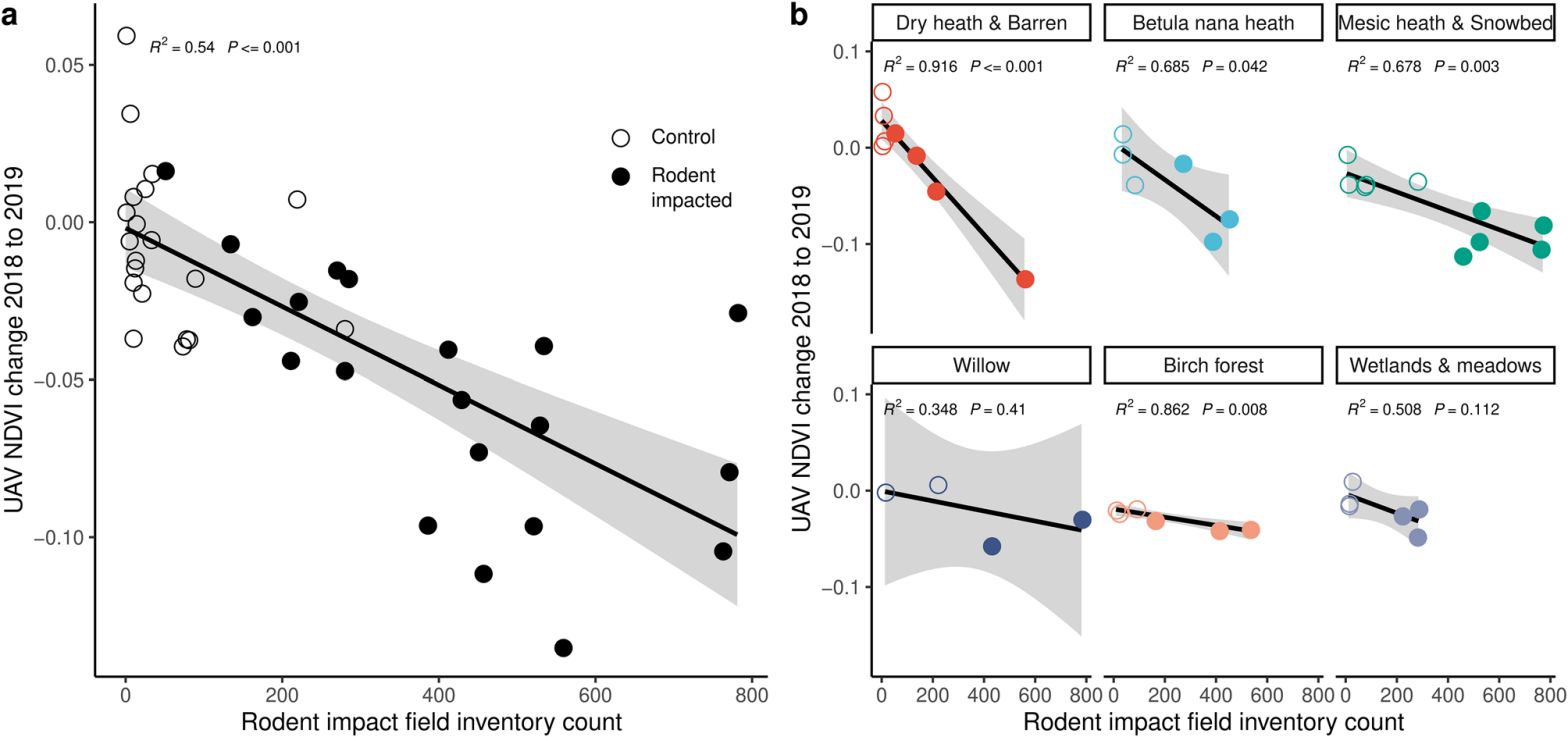
(**a**) Relationship between rodent impact field inventory count in 2 m × 2 m field plots and UAV derived NDVI change for all habitats combined (r^2^ = 0.54; p =  < 0.001; grey shadow = 95% CI). (**b**) Comparison of rodent impact count in 2 m × 2 m field plots for different habitats. Dry tundra and barren, as well as wetland and meadow habitat types were aggregated.

In this article, we aim to provide a novel understanding on the spatial dimension of herbivory, and highlight the substantial potential of UAVs to address important knowledge gaps in plant herbivore interactions. We used repeated UAV flights equipped with RGB and multispectral sensors in four 15–21 hectare large areas during (2018) and after (2019) a vole and lemming peak in the tundra in northernmost Sweden to survey the impact of rodent peaks with a high spatial resolution at landscape scale (Fig. 1). We verified that the detected changes in vegetation productivity were indeed due to vole and lemming impact through visual comparison of RGB and multispectral UAV imagery from 2018 to 2019 (Fig. 1), and by comparing the estimated impact based on multispectral data with field recorded impact in ground truth investigations (Fig. 2; Supplementary Fig. S2). Our ground truth inventory recorded traces of vole and lemming activity apparent as bite marks in vegetation, clipped vegetation remains, droppings, burrows or runways^31^. The impact is visible in the field as missing or dead vegetation, leading to browning of the surface (Fig. 1) and reduced vegetation productivity when measured using the NDVI in the field, from satellites^10^ and in our UAV imagery (Fig. 1). We observed rodent nest sites covering patches of 0.3–1 m diameter associated with burrows and cavities and cleared of vegetation. Runways that are used for movement under snow, were visible as linear features in the UAV imagery. We also observed extensive impact where low growth tundra vegetation has been thinned out over large areas (Fig. 1, Supplementary Fig. S3). Only inventoried impacts that were clearly attributable to voles or lemmings were included as control data. Other vegetation damage, such as reindeer herbivory or fungal die-off, were not included as rodent impact in our ground control (Fig. 2). Further, we mapped habitats at each study area using an object-based land cover classification approach appropriate for complex Arctic terrain^18,32^.

Our results show, that the field inventory of rodent impact was linearly correlated with NDVI change between 2018 and 2019 in ground control plots (Fig. 2a, r^2^ = 0.540). Both the r^2^ values and slope estimates differed between habitats and was highest in the low productive and structurally simple heath habitats, and lower in the more complex willow, forest and wetland patches (Fig. 2b). We mapped rodent impacted areas in the UAV imagery in separate best estimate, minimum and maximum scenarios. The impact was mapped by applying a threshold of negative NDVI change between two years and masking of habitats. The resulting maps aligned well with our field inventory count of rodent impact in our control plots (Supplementary Fig. S2). However, the traditional ground truth estimate of herbivory impacts by counting impacted quadrants that we used^31^ is not an exact quantitative measurement of rodent impact either, but the strong relationship between different measures (UAV and ground truth) supports that both methods successfully estimate comparable key aspects of vole and lemming impacts but in different ways.

**Figure 3:**
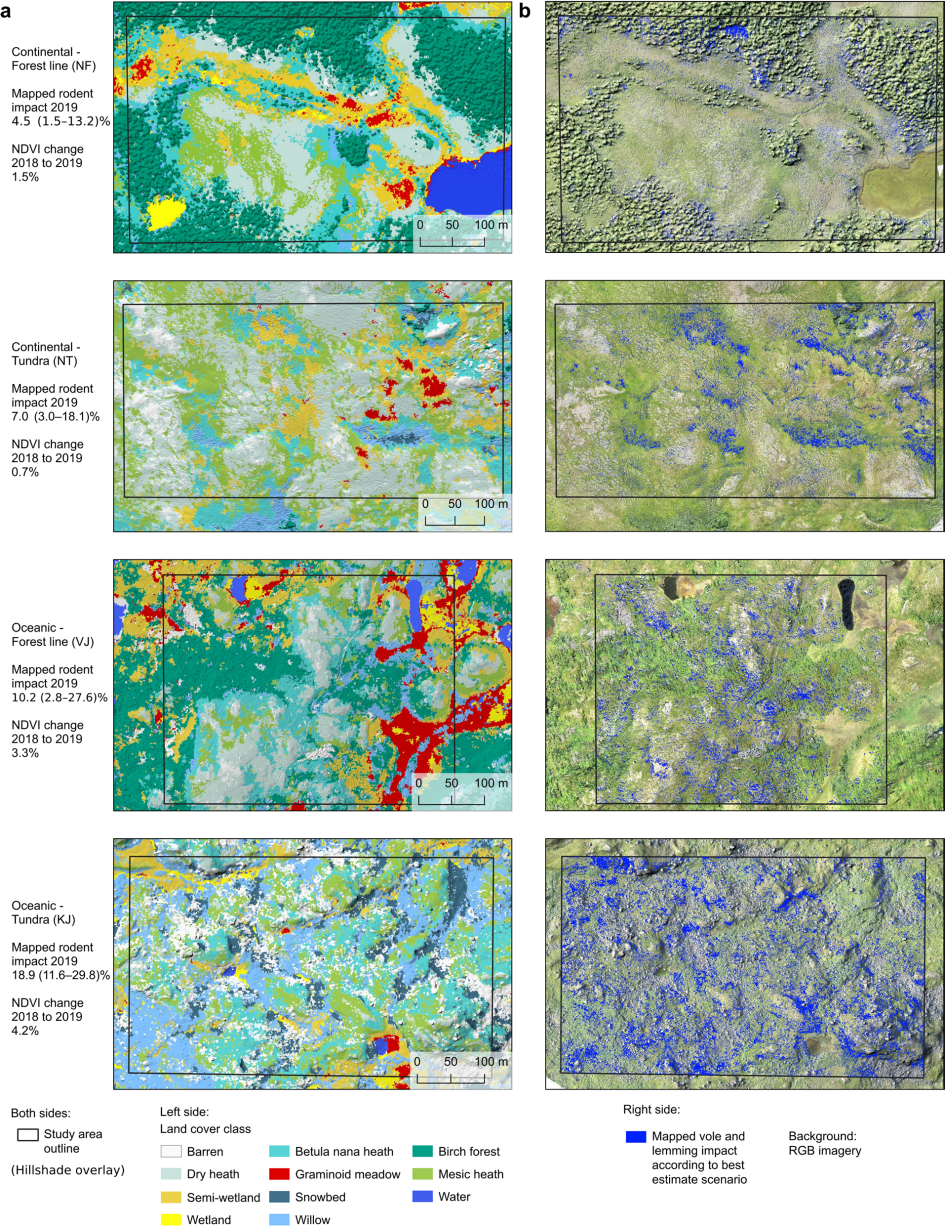
(**a**) Habitats (land cover classifications) for each study area. (**b**) Mapped vole and lemming impact according to the best estimate scenario (blue) over truecolor RGB UAV imagery. Figure created in QGIS 3.18 (www.qgis.org).

Our long-term rodent trapping time-series indicates a rodent peak in autumn 2018 (Fig. 1c, Supplementary Fig. S1)^22^ and the absence of a lemming peak. During the past two decades, vole populations have fluctuated in 3-to-5-year population cycles, with synchronous lemming peaks accompanying some of the vole peaks (Supplementary Fig. S1)^23^. The peak in 2018 was the lowest during the whole time-series (Fig. 1c) and no lemmings were trapped (Supplementary Fig. S1), although winter nests, dead lemmings and feeding marks on vegetation indicate that lemmings were detected at least in the KJ site. In general, the high impact of rodents is known to happen during the autumn and winter after peak densities are recorded in the trapping, and thus observed the following year in the vegetation^23,24,33,34^. The fairly small vole and lemming peak in 2018 thus resulted in a relatively small reduction in plant biomass (4–16%), GPP (3–12%) and NDVI (0.7–4%) compared to previous peaks during the last two decades (Table 1)^23^, but rodents still impacted between 4.5 and 18.9% of the landscape according to our best estimate from the UAV data (Fig. 3, Table 1). This supports previous studies identifying voles and lemmings as drivers of Arctic vegetation^23,33,35–37^, and indicate that even small peaks have substantial impacts on ecosystems although it would be hard to record using traditional methods linking field plots or satellites^23^.

**Table 1:** Summary of mapped rodent impact, the effect on NDVI and parameters of spatial autocorrelation at study area level and the estimated effect this has on GPP and biomass.

Study area	Type	Mapped rodent impact 2019 (%) Best estimate (Min.–Max.)	Mean NDVI 2018	Mean NDVI 2019	Change from 2018 to 2019				Spatial autocorrelation of rodent impact	
					NDVI	NDVI (%)	GPP (%)	Biomass (% )	Moran's I	Max. variogram range (m)
NF	Continental forest line, low altitude	4.5 (1.5–13.2)	0.693	0.682	− 0.011	− 1.5	− 5.1	− 6.6	0.56	4.3
NT	Continental tundra, high altitude	7.0 (3.0–18.1)	0.610	0.606	− 0.004	− 0.7	− 3.0	− 4.2	0.62	4.2
VJ	Oceanic forest line, low altitude	10.2 (2.8–27.6)	0.701	0.678	− 0.023	− 3.3	− 9.1	− 11.3	0.61	3.3
KJ	Oceanic tundra, high altitude	18.9 (11.6–29.8)	0.628	0.601	− 0.027	− 4.2	− 12.4	− 15.7	0.66	3.5

**Figure 4:**
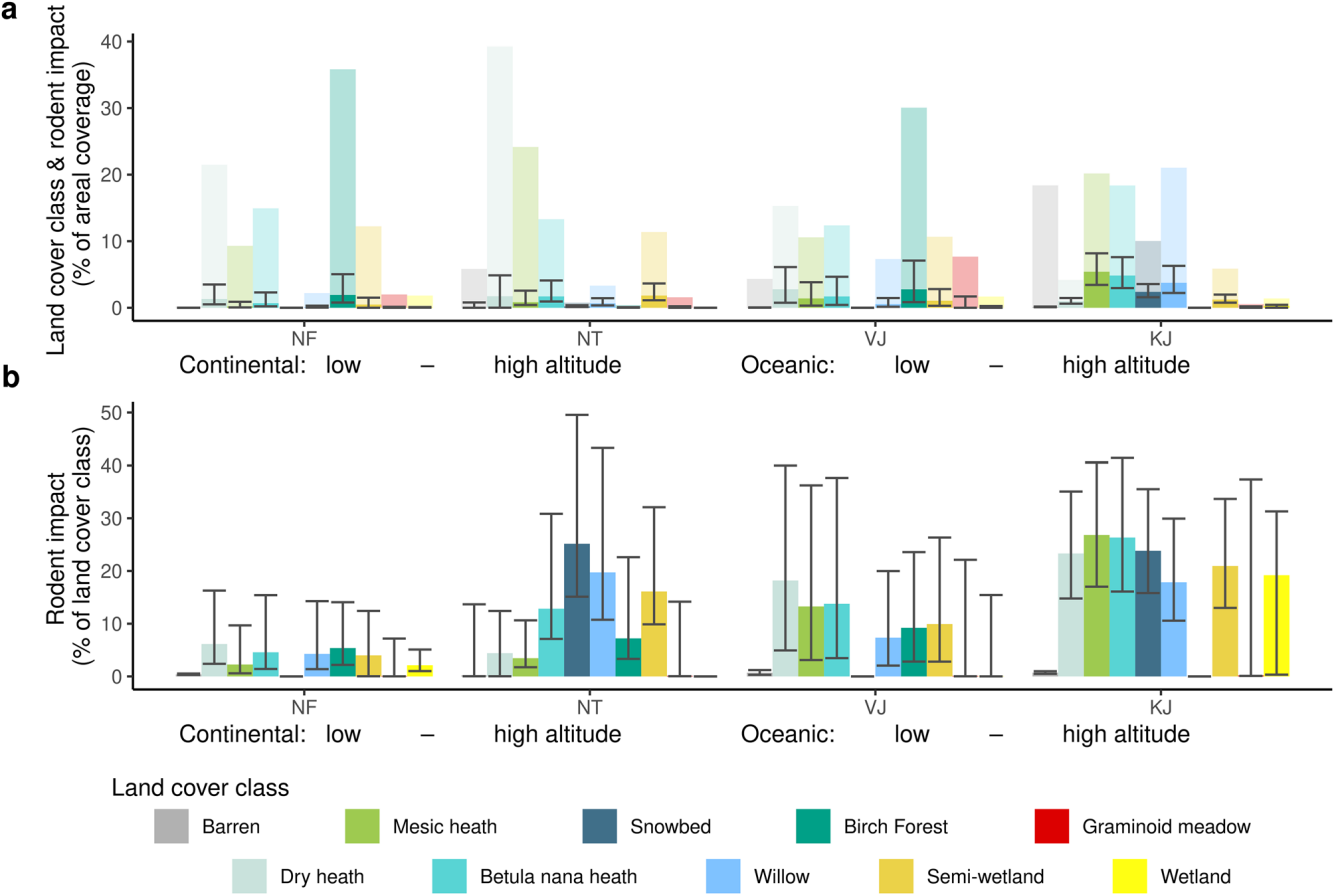
(**a**) Mapped land cover area for each study area (background bars) and rodent impact for the best estimate (full color bars), minimum and maximum (error bars) scenarios (% of total area excluding water). (**b**) Rodent impact fraction (%) within each respective land cover class for the best estimate (full color bars), minimum and maximum (error bars) scenarios.

Furthermore, we can quantify the spatial heterogeneity of the rodent impact across scales. At regional scale, across all four study areas, the mapped rodent impact was higher in the oceanic locations than in the continental and in the high-altitude sites (NT, KJ) than in the low altitude sites (NF, VJ; Fig. 3; Table 1). The higher impact levels in oceanic (VJ, KJ) than in continental locations (NF, NT) are supported by higher rodent densities in the oceanic region than in the continental region (Supplementary Fig. S1) and could be linked to in average deeper snow in most habitats. This maintains a larger fraction of the landscape as a snow covered and suitable habitat at local scale, creating more favourable conditions for rodents during winter and extended snow seasons^17,38^. The higher impact in high altitude sites does, on the other hand, support previous studies proposing that herbivory impact in the tundra is higher in high altitude sites with typically lower predator densities than in low altitude sites where predators thrive^12,39^. At local scale, the level of mapped rodent impact differed across the different habitat classes within each location (Fig. 4). The moist and fairly snow-rich *Betula nana* heath, willow shrubs, snowbeds and semi-wetlands were heavily impacted in all locations while barren habitats and graminoid meadows were lightly impacted in all locations reflecting often poor snow conditions. Dry heath and mesic heath habitats experienced contrasting rodent impact among locations with a low impact in the most snow poor location (continental tundra) and high impact in the typically snow rich oceanic locations. These habitats are probably below the snow threshold to be suitable winter conditions for voles and lemmings in the continental tundra location^24^ where we rarely observe more than 10 cm of snow cover during winter^23^, while the snow cover might be enough at the snow-rich oceanic locations. The impact in the birch forest is mixed. This might be the result of a heterogeneous habitat with dry and wet vegetation or linked to methodological aspects in detecting rodent impact in complex habitats (Fig. 2b).

Although, these results correspond with previous findings in descriptive and experimental studies^23,24,40–42^, this is the first time the heterogeneity of the impact has been quantified at this large spatial scale, which is a prerequisite for incorporating the results in earth ecosystem models or other large-scale upscaling approaches^43^ in a sophisticated way. At habitat scale, the rodent impact was highly aggregated even at pixel resolution (11–13 cm). This is evident from high Moran’s I (> 0.5; Table 1), the range > 4.5 m for spatial-autocorrelation in variograms of rodent impact (Supplementary Fig. S4, Table 1), and the heterogeneous impact even within habitats (Fig. 3). This is the first time the heterogeneity of the impact of herbivores has been quantified at such detail across such large areas and reveals that the heterogeneity is large enough to matter for the way we can analyse the effect of herbivores on ecosystem structure and function using remote sensing products^17^. Albeit tools like GPS collars have resulted in rapid advances in movement ecology^44^, just data on the activity of the herbivores does not reveal their impact on the vegetation. Quantifying the heterogeneity of the impact of herbivores on ecosystems is very important since it will influence the heterogeneity of landscapes and thus species diversity^15^, ecosystem stability^16^ and a resistance to effects of a warming climate^7^.

This study gives a new perspective on the impact by rodents on Arctic ecosystems. Even this small rodent peak, the by far weakest during the last decades (Fig. 1c), caused a decrease in NDVI of 0.7–4.2% at landscape scale and thus comparable in effect size to other events considered highly important^45^, severely impacted up to 19% of the landscape (Fig. 4, Table 1), and had even more pronounced effects on certain habitats (Fig. 4). Given that vole and lemming populations experience population fluctuations across the Arctic^22^, rodent peaks should be important disturbance events in almost the entire biome. The slow growth rates of low productive Arctic vegetation^23^, result in the Arctic tundra being a mosaic of patches in different successional stages with implications for plant community composition and plant nutrient content^36^. Quantifying the heterogeneity of rodent impacts using UAVs makes it possible to estimate the importance of rodents on fundamental aspects of Arctic ecosystems, i.e. productivity, carbon storage, biodiversity and responses to a changing climate. The productivity of the tundra at panArctic scales is often estimated through increases in NDVI (spectral greening; increased productivity) or decreases (browning; decreased productivity)^45^, since NDVI and other indexes derived from satellites correlate well with gross photosynthesis^46^. Although the greening trends are known to be heterogeneous among regions and habitat^45^, our results record a high heterogeneity even at sub-meter scale (Fig. 1) and spatial autocorrelation with a range > 4.5 m (Supplementary Fig. S4). This is important since it means that the greening or browning of satellite pixels (10–500 m) is the result of heterogeneous changes at subpixel scale, and that the configuration of environmental conditions and rodent disturbance might be responsible for changes in spectral greening or browning rather than pixel means, hence compromise an important scaling effect^17^. Since the relationship between gross primary production and NDVI is nonlinear, determining the effect of a heterogeneous impact like rodent peaks will severely underestimate their importance on ecosystem productivity if this heterogeneity is not estimated and considered (see Siewert and Olofsson^17^ for an in depth explanation of this scaling mechanism). This mosaic of areas in different successional stages of vegetation is expected to strongly promote species diversity by preventing competitive exclusion and by inducing environmental heterogeneity^15^. Finally, the mapped heterogeneity hints at a new perspective on how these ecosystems may respond to a given climate scenario. A changing climate is expected to result in changes of the population dynamics^22^ as well as habitat selection^47^ of voles and lemmings. Our results are consistent with previous conclusions that crashing vole and lemming populations due to warm winter conditions in the southern edge of their distribution range could drive large ecosystem changes in the future^48^, and that changes in their behaviour and habitat choice influence land cover heterogeneity and thus structure and function of Arctic ecosystems well before population crashes are detected. Such dynamics are presently not integrated in major Arctic ecosystem assessments, but UAV data can clearly help to integrate this.

Our estimate shows very good agreement with ground control data (Fig. 2, Supplementary Fig. S2) and demonstrates the potential of UAV imagery to generate a better understanding of impacts of herbivory at the scale of the process. Further, long-term monitoring may provide insights at landscape scale without disturbance by human presence, that would previously have been restricted to elaborate field campaigns or large scale controlled experiments^39^. However, our analysis is also facing a few uncertainties. For capture and processing of the UAV imagery, we harmonized relevant factors, e.g. weather conditions, programmed flight patterns and processing presets whenever possible and following best practices^29^. UAV maps are composites of several hundred images leading to minor differences in geometric positioning of pixels between years (i.e. co-registration) and artefacts in change detection which risks overestimation of the rodent impact. Weather and logistical constraints prohibited identical light conditions between years for all areas while environmental conditions change naturally. The mapped and ground-truthed rodent impact was not directly proportional to the reduction in NDVI across all four study sites, indicating that other factors also influenced the NDVI at this scale. Yet, the highest impacted area (KJ: 19%), also had the strongest reduction in NDVI (− 4.2%), GPP (− 12.4%) and biomass (− 15.7%). The relationship of NDVI with derived GPP and biomass is subject to a number of assumptions, and to some extent habitat and location specific^17,45,46^. Environmental factors leading to potential under- or overestimation may include inter-annual differences in vegetation productivity, seasonal variation of water ponds or surface wetness at the time of the UAV flight. Climate and season length were fairly similar both years 2018 and 2019 compared to for instance 2017 and 2020. Further, the impact of rodents is strongest in the field layer of vegetation, but in complex habitats, this may be obscured in the aerial view under canopy-forming vegetation including willow or birch tree patches. Our method using a threshold of NDVI reduction is only sensitive when this threshold is met thus risking underestimation. The complexity of the terrain offers a varied insight into herbivory dynamics, but increases the challenge to generate land cover maps showing habitats. We addressed these uncertainties with best estimate, minimum and maximum scenarios that we generated using different thresholds, and masking of certain habitats. As the same thresholds are applied per study area, the rodent impact might be over- or underestimated in the different habitats depending on environmental conditions, but we are confident that the estimates are fairly accurate, shown by the close correspondence to ground control estimates (Fig. 2, Supplementary Fig. S2), and that the real rodent impact is within the range of these scenarios.

The low productivity and low statured vegetation^17,29,49^ combined with the distinct population peaks of the major herbivores^22^ make the Arctic tundra especially suitable for quantifying the heterogeneity of herbivore impact using UAVs. The results indicate that large areas are impacted even during a small rodent peak. Mapping the extent and heterogeneity of the impact by rodents at large spatial scales is a vital step to produce results that can be incorporated in scenarios or ecosystem models and thus fully accounting for this process in our understanding of ecosystem changes and ecosystem projections. In general, we consider UAVs to be a very promising method to understand key herbivory and ecosystem dynamics, especially when combined with tools such as exclosures, GPS collars and satellite images.

## Methods

### Study area

Four areas with an extent of 15–21 ha (~ 350 m ×  ~ 500 m) were studied in a paired setup in the Torneträsk region, northern Sweden. The setup covers at large the climatic variability of the Fennoscandian forest-tundra ecotone. Two continental sites are situated near the Nissonjokka gorge about 10 and 15 km southwest of Abisko (Nisson forest; NF; 525 m.a.s.l.; (68° 19′ N, 18° 49′ E) and Nisson tundra; NT; 690 m.a.s.l.; 68° 18′ N, 18° 50′ E). The mean annual air temperature (MAAT) was − 0.8 °C for the 1961–90 reference period, while mean annual precipitation (MAP) was 304 mm for the same interval (Abisko Scientific Research Station). Two sites with an oceanic climate (1961–90: − 2 °C MAAT; 844 mm MAP; Kåtterjokk) are situated east of lake Vassijaure (VJ; 490 m.a.s.l. 68° 26′ N, 18° 15′ E) and 1.5 km south of Katterjokk (KJ; 600 m.a.s.l; 68° 24′ 43″ N, 18° 08′ E). The treeline is formed by mountain birch (*Betula pubescens *ssp.* czerepanovii*) forests as is typical for Fennoscandia. The lower altitude sites (NF, VJ) feature patches of open heath tundra, mountain birch forest and wetlands. The higher altitude sites (NT, KJ) are dominated by open heath tundra and barren terrain interrupted by shrub, snowbed communities, graminoid meadows and minor mountain birch stands. Bedrock is nutrient-poor in all four areas. Dominant species for heath communities are *Vaccinium vitis-idaea*, *Vaccinium myrtillus*, *Empetrum hermaphroditum* and *Betula nana.* Dense shrub communities are formed by *Salix* spp. and *Betula nana.*

### Rodent field inventory

Vole and lemming population densities were monitored separately for the continental and the oceanic region in spring and fall since 1998^23^. The catch rate reflects individuals per 100 trap nights using the small quadrat survey method^23,50^. In 2019 during the early vegetation season (June–July), we inventoried rodent impact on vegetation and traces in five paired ground control plots (2 m × 2 m) per study area totalling 40 plots. These were laid out in selected heavily grazed (*impacted*) and relatively ungrazed (*control*) areas. Plot pairs were only set in a subset of the most representative habitats due to the labour intensity of the method. Rodent herbivory was recorded in a raster of 0.1 m × 0.1 m quadrants within these plots, approximating the resolution of the UAV imagery (400 total quadrants per plot). Observed impact was categorized in vegetation bite marks, feces droppings, runways and burrows. Each impact was given 1 point with multiple impacts possible per quadrant. These were then summed per plot. All ground plots were georeferenced using DGPS. For the impact inventory, this was controlled using markers at 1 m distance to the plot visible in the UAV imagery. This allowed to extract the NDVI from the UAV imagery on a plot basis.

### UAV data

We recorded UAV imagery using an *eBee* fixed-wing mapping drone (senseFly, Switzerland) with *Sequoia* multi-spectral (Parrot, France) and G9X RGB (Canon, Japan) cameras. For the assessement of rodent impact, imagery was recorded during vegetation peak productivity between July 27th and August 4th in 2018 and 2019. Additional imagery from right after snowmelt in June and in September was used to construct land cover classifications. The *Sequoia* sensor provides imagery in green, red, red-edge and near-infrared bands (G, R, RE, NIR). The normalized difference vegetation index (NDVI) was extracted using the R and NIR bands. Radiometric calibration was performed using spectral reflectance panels (MosaicMill, Finland). The multispectral imagery had a ground resolution of 11.4–13.5 cm depending on flight and area after photogrammetric processing using *Pix4Dmapper* (Pix4D SA, Switzerland). The RGB imagery had a resolution of (1.5–3.3 cm). The UAV imagery was georeferenced with 4–8 ground reference markers using centimeter precision DGPS. Co-registration of the multispectral UAV imagery between 2018 and 2019 was generally near pixel-perfect and did mostly not deviate more than 2–3 pixel for locations within the analyzed core mapping area. Fine-registration algorithms available in Orfeo Toolbox v. 7.1^51^ and RStoolbox^52^ were tested, but did not further improve the co-registration and were thus not applied.

### Land cover maps

Object-based land cover maps reflecting different ecological habitats were generated for each study area as is appropriate for highly heterogeneous Arctic study areas^18,32^. First, the area was segmented based on four UAV bands (G, R, RE, NIR) at a resolution of 0.3 m using mean shift segmentation from Orfeo Toolbox v. 7.1^51^. Environmental data to inform the classification was extracted from early seasonal, peak seasonal and late seasonal UAV flights of 2018 using the spectral bands and the NDVI. Additional topographic layers were generated either from the UAV imagery or the Swedish national elevation data at 2 m resolution or laser data (Lantmäteriet). These additional layers were vegetation height, catchment slope, wetness index (SAGA)^53^, relative slope position and slope. Based on the environmental data, the segments were then classified using a random forest machine learning classifier^54^. Training areas were identified in the RGB UAV imagery and on field photographs. The classes describe common Arctic land cover types and reflect potential vole and lemming habitats (Supplementary Methods for a description). Accuracy was assessed using the out-of-bag error, Overall Accurcay and the Kappa index (Supplementary Discussion). Rodent impact was subsequently extracted per land cover class.

### Rodent impact mapping from UAV imagery

Rodent impact in the UAV imagery can be identified as change from the 2018 to the 2019 imagery. Many change detection methods exist in remote sensing^55,56^. We reviewed and tested several methods to map the impact of rodents on vegetation. The visually detectable impact in the UAV imagery is as fine-grained as the pixel resolution. Therefore, only pixel-based and no object-based change detection methods were considered. The tested methods included *raster math* (also termed *image differencing)*, *principal component analysis* (*PCA)*, *change vector analysis (CVA)*, direct *binary classification using machine learning* and *multivariate alteration detector (MAD)*^55,56^. These were applied to either the full set of multispectral bands (G, R, RE, NIR) or to the extracted NDVI imagery. After initial results we selected *raster math* subtracting the 2019 NDVI imagery from 2018 NDVI imagery as the best performing method. Other results are not presented. The selection criteria were visual detection of rodent impact in agreement with the higher resolution RGB imagery comparing 2018–2019, and robustness against artefacts and overestimation. The 2018 NDVI imagery was resampled to match the raster grid of the 2019 imagery using the nearest neighbour algorithm. This improved the co-registration of the imagery and reduced artefacts in the final result.

The *raster math* method provided maps showing quantitative changes in NDVI from 2018 to 2019. A threshold of reduction in NDVI was selected to subset the maps to reflect rodent impacts. At known field locations with photographs and from the RGB imagery, we observed areas of intensive impact, such as major vegetation grazing, nests and runways clearly identifiable as brown patches replacing previously intact vegetation cover, but also extensive impact such as individual clipped plants thinning out the vegetation cover (Fig. 1d, Supplementary Fig. S3). The change detection was sensitive to artefacts from photogrammetric processing, changing light and moisture conditions between flights. To accommodate for this, we applied three impact scenarios of best estimate, minimum and maximum rodent impact for each area. The selected threshold varied between study area and scenario (− 0.04 to − 0.1 NDVI). Further, we used the land cover classification to mask different habitats in which we assumed mapped impact to be a potential artefact or overestimate, again depending on different scenarios.

We also extracted changes in NDVI, standing biomass and gross primary productivity (GPP) from 2018 to 2019 as well as mapped rodent impact (best estimate scenario) as mean per study area. Biomass and GPP were derived by applying regression formulas following Siewert and Olofsson^17^.

Spatial autocorrelation was measured by calculating Moran’s I using the *R ‘raster’* package^57^ and variograms using the *‘gstat’* package^58^ from the rodent impact best estimate raster maps. Values for Moran’s I range between − 1 to 1 with values close to 1 indicating strong autocorrelation and clustering, − 1 indicating perfect dispersal and 0 indicating spatial randomness^59^. Experimental variograms were generated by drawing a random sample of 100,000 pixels of the raster maps and a width of 0.3 m. Different variogram models were tested and a double exponential term proofed to provide the best approximation.

## Supplementary Information

Supplementary Information.

## Data Availability

All data handling, processing and statistical analyses was performed using R statistical software^60^ unless mentioned otherwise. Data was visualized using R^60^ or QGIS^61^. The code for this study, supporting table and vector data is available from the code repository of the corresponding author. The UAV NDVI orthomaps and the land cover classification data will be made available through the PANGAEA scientific repository (https://pangaea.de/) upon publication and will be available through the search function in the repository. The UAV RGB orthomaps are available upon reasonable request.
