# Supplementary material for: UAV reveals substantial but heterogeneous effects of herbivores on Arctic vegetation
Source: Sci Rep. 2021 Sep 30;11:19468. doi: 10.1038/s41598-021-98497-5 (PMC8484448; doi:10.1038/s41598-021-98497-5)
Supplement: Supplementary file 1 — Supplementary Information. [file 41598_2021_98497_MOESM1_ESM.pdf]

Supplementary information

UAV reveals substantial but heterogeneous effects of herbivores on Arctic vegetation

Matthias B. Siewert[1*], Johan Olofsson[1]

[1]Department of Ecology and Environmental Science, Umeå University, Umeå, Sweden

*Email: matthias.siewert@umu.se

Supplementary Figures

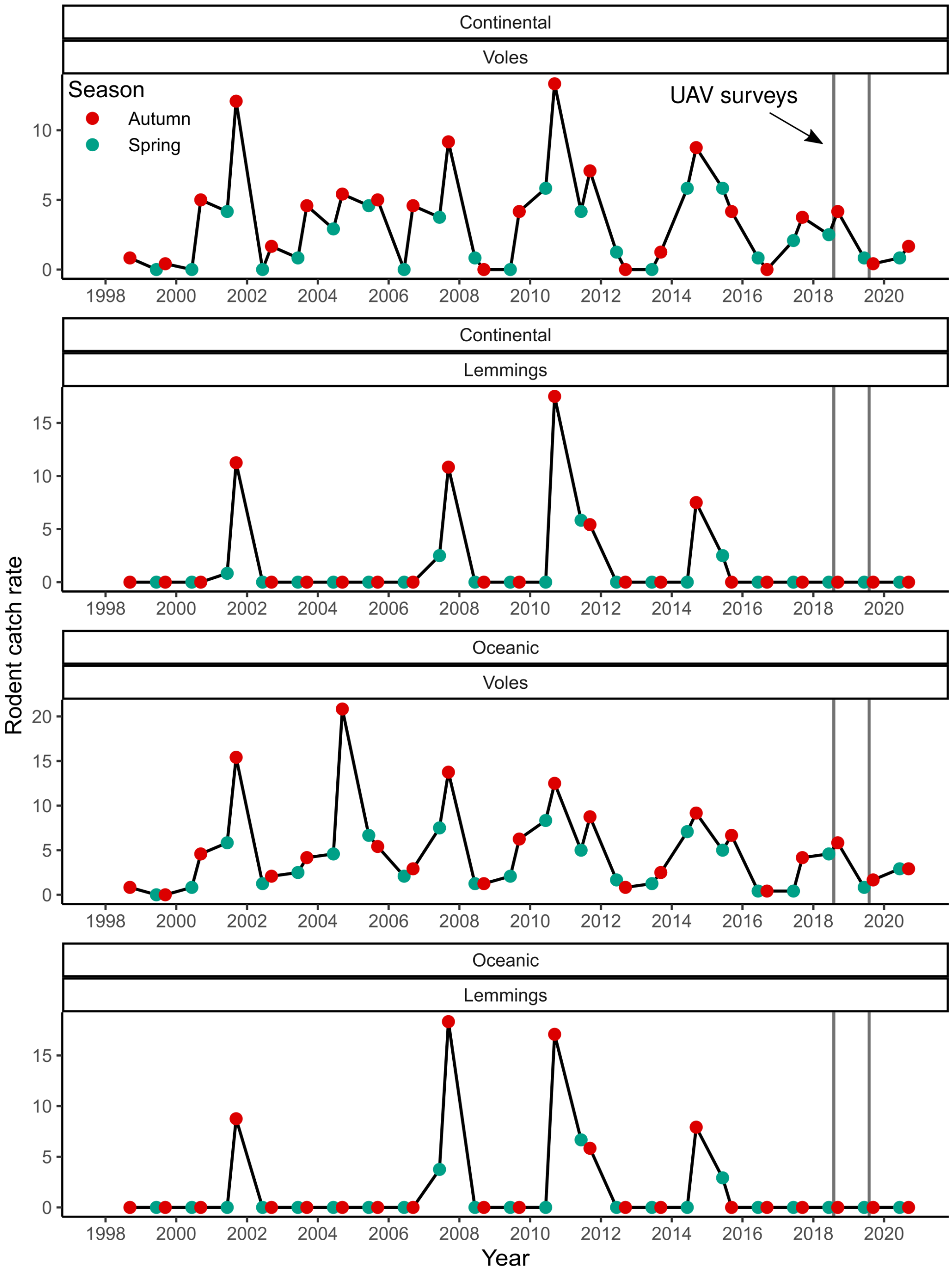

Figure S1| Vole and lemming catch rates for 1998 to 2020 differentiated for voles and lemmings, as well as oceanic and continental areas. The vertical lines indicate time of UAV flights in summer 2018 and 2019.

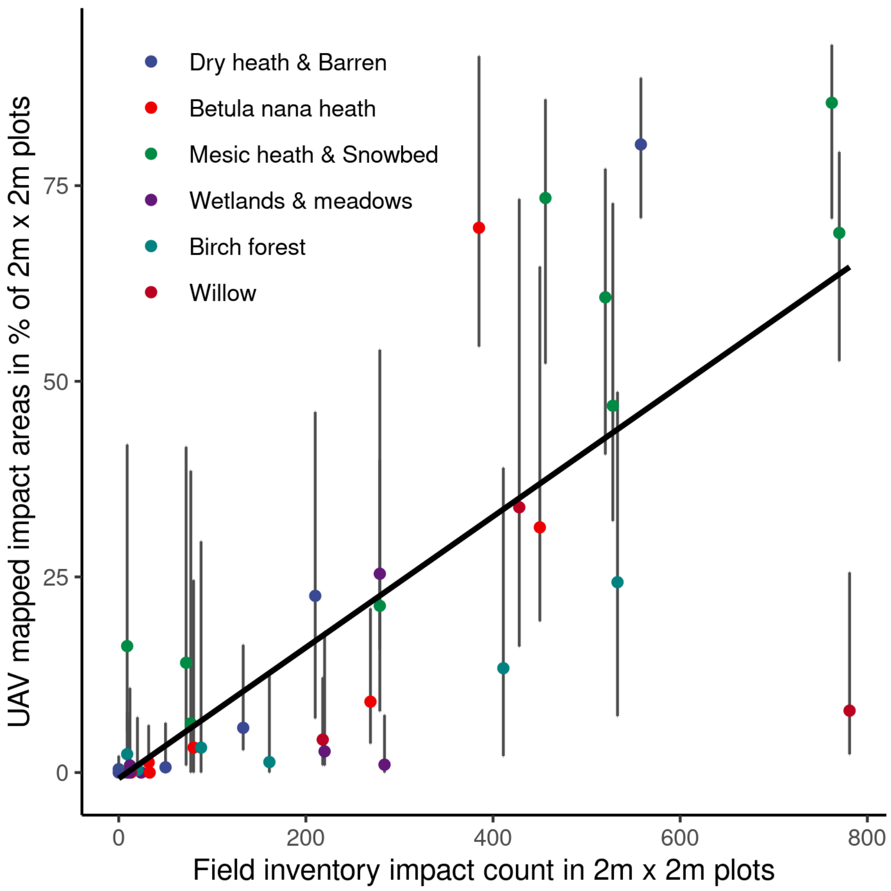

Figure S2| Fit between field inventoried rodent impact (count) and UAV mapped rodent impact (% cover) for 2 m ×2 m impact plots. Each point represents one plot. Error bars represent the minimum and maximum scenario from the UAV inventory.

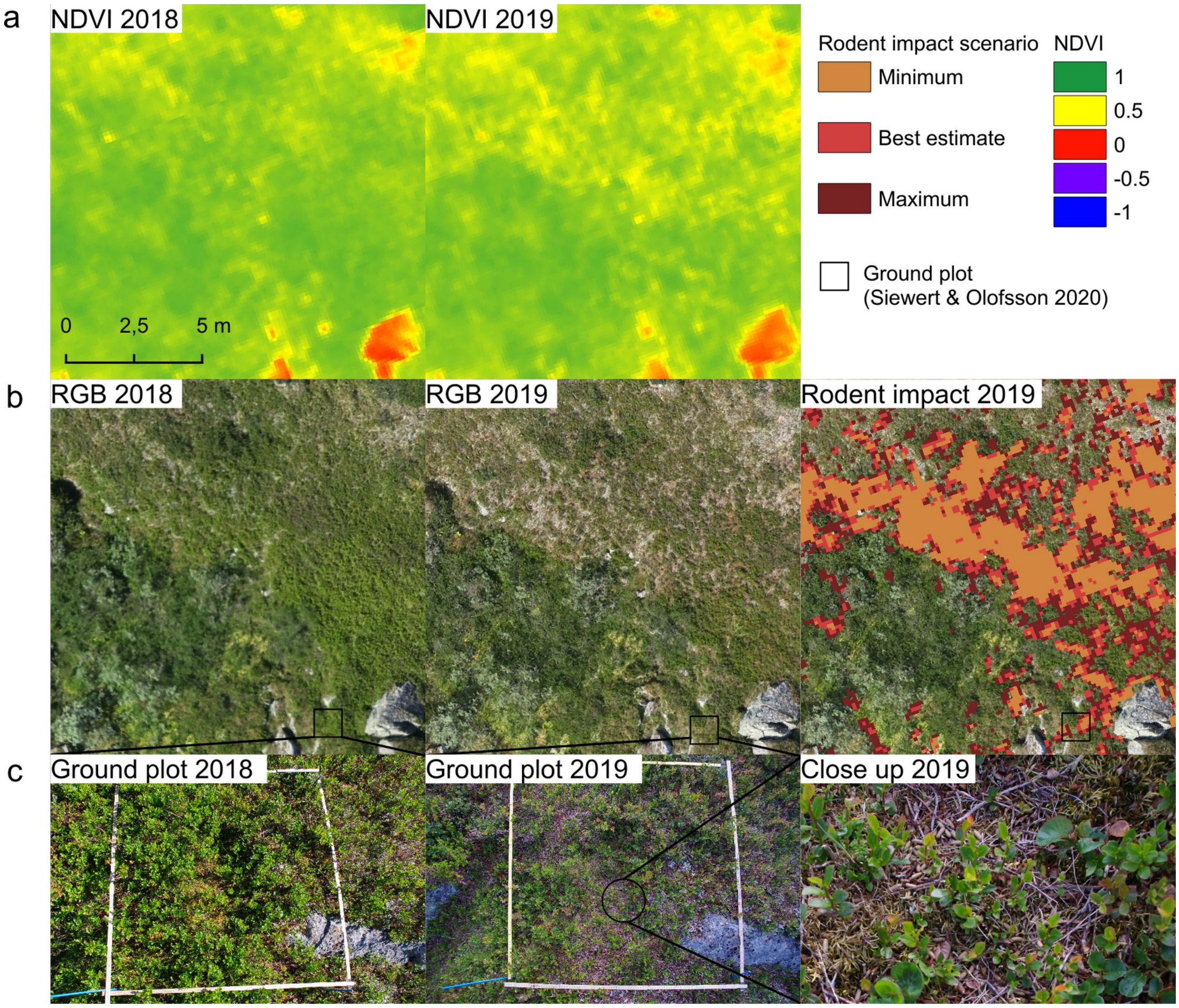

Figure S3| Example of heavily grazed area at the oceanic high altitude site (KJ). a. UAV NDVI imagery for 2018 and 2019. b. UAV RGB truecolor imagery for 2018, 2019 and 2019 overlayed with 3 rodent impact scenarios. c. Photographs from 2018 and 2019 and close-up of clipped vegetation and rodent feces dropings from a permanent ground plot.

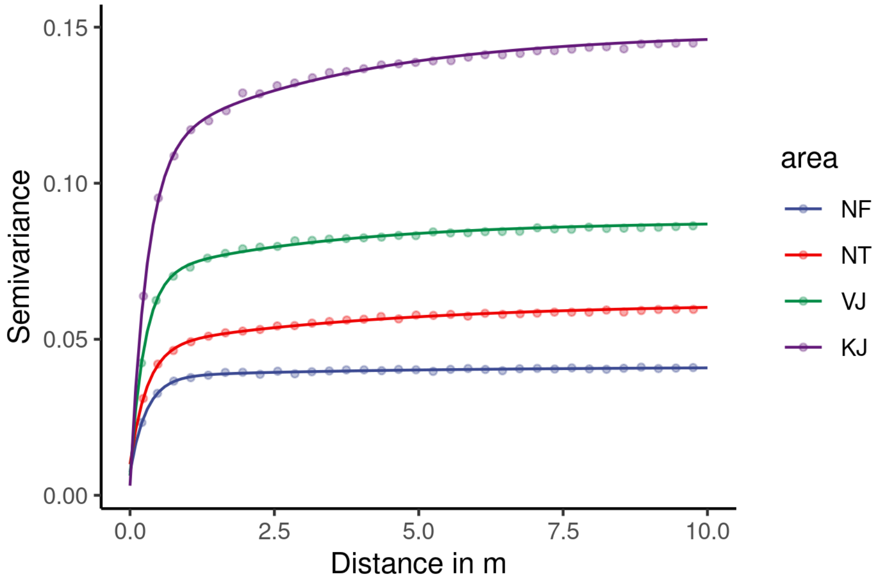

Figure S4| Variograms of UAV mapped vole and lemming impact in the best estimate scenario. Variogram models were approximated using a double exponential term revealing spatial autocorrelation up to 3.3 – 4.3 m depending on the area.

Supplementary Methods

Description of land cover classes

**Class name**	**Class description and key properties**	**Example photographs (with name of ground plots from Siewert & Olofsson[1])**
Barren	Rocks, boulders, barren and sparsely vegetated areas with <10-20% vegetation cover consisting for example of *Vaccinium uliginosum* or *Empetrum hermaphroditum* patches, ground can be covered by lichen crusts or soil.	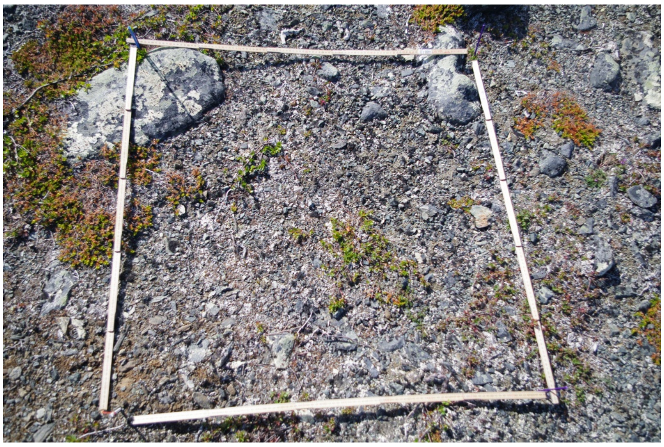<br>NT-14
		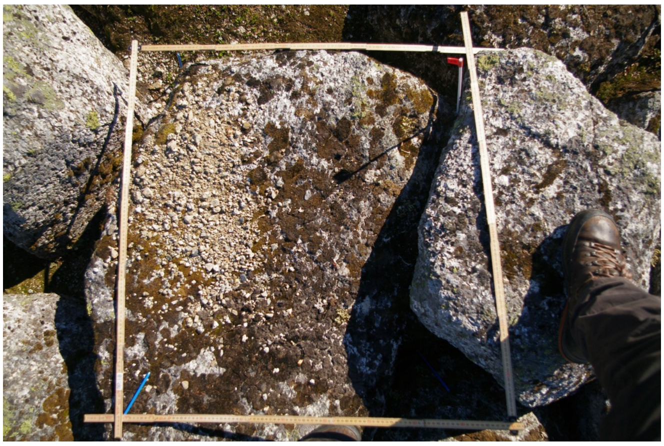<br>KJ-24
Dry heath	Alternating vegetated patches of mesic heath and barren ground. Associated with patterned ground and solifluction lobes. Mostly dominated by *Empetrum hermaphroditum*, *Betula nana or Vaccinium uliginosum.*	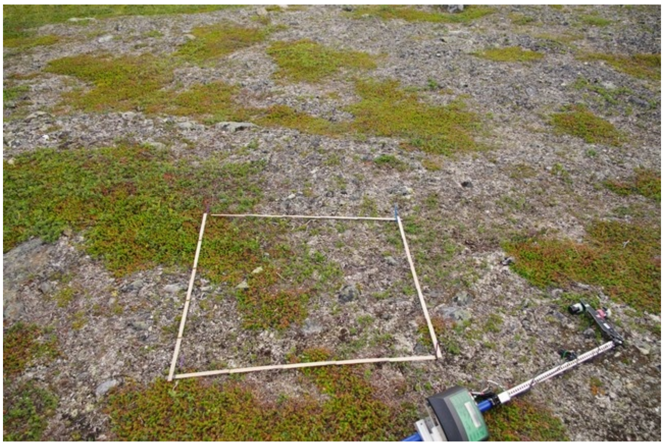<br>NT-20

		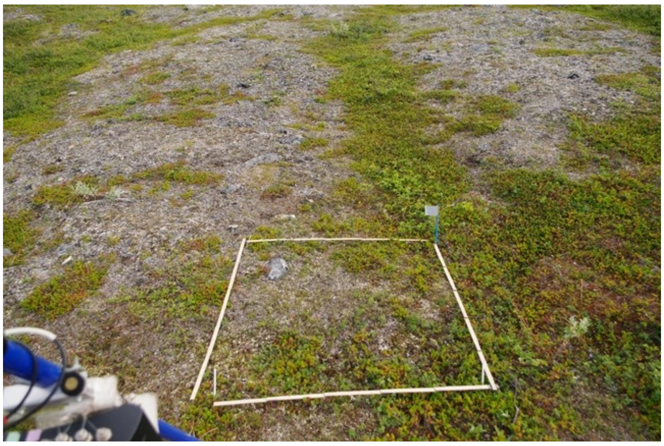 NF-06
		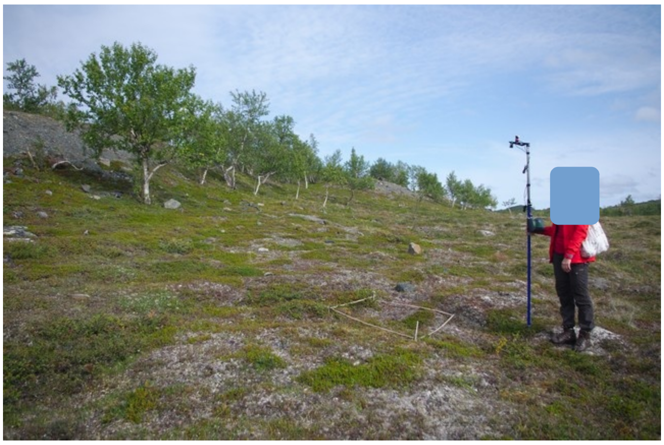 NF-1
Mesic heath	Heath of intermediate moisture defined by a continuous low growth vegetation cover. Often dominated by *Empetrum hermaphroditum*, with *Betula nana*, *Vaccinium vitis-idaea*, *Vaccinium myrtillus*, but also mossy or herbaceous vegetation.	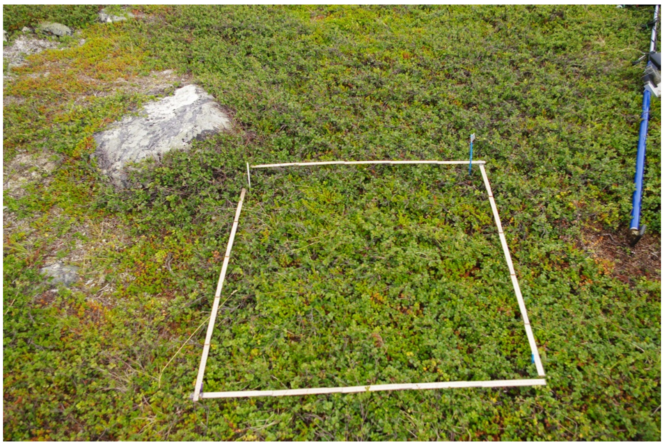 NT-21
		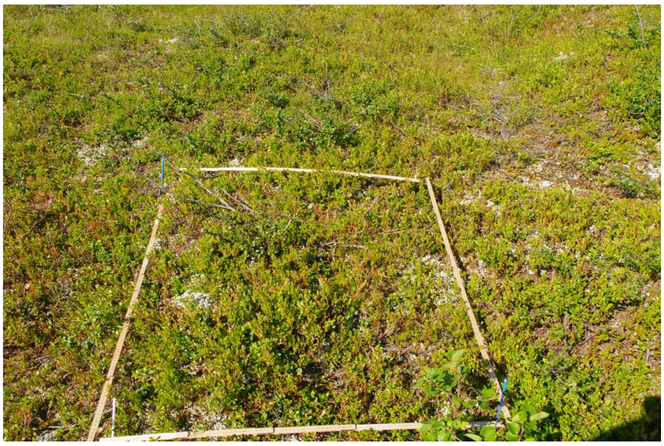 VJ-05

		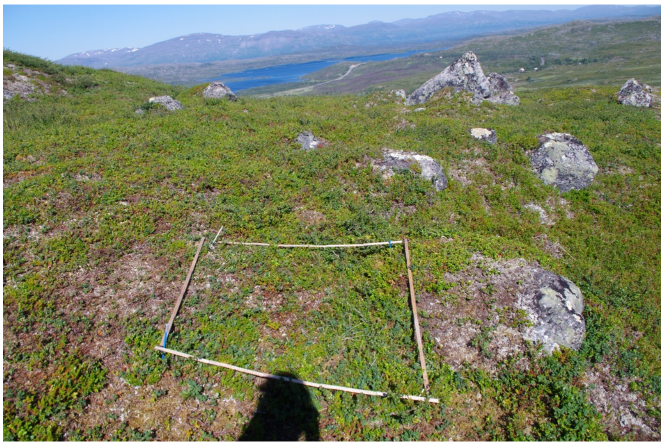 KJ-13
Betula nana heath	Betula nana dominated heath, associated with *Empetrum hermaphroditum* understory and individual *Salix spp.* shrubs. Often a transitional land cover to dry heath and mesic heath, as well as to willow shrub communities and mountain birch forest.	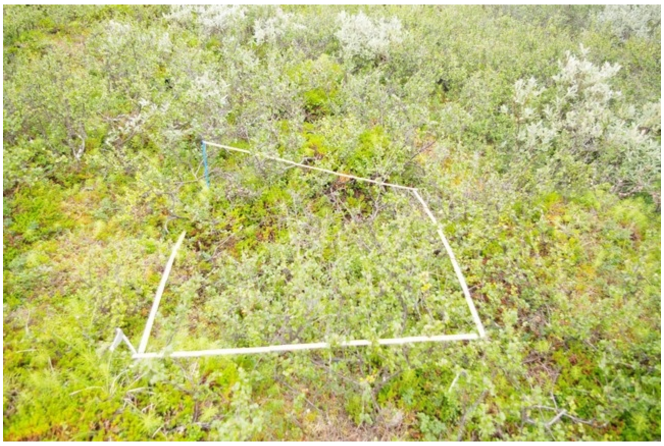 NF-13
		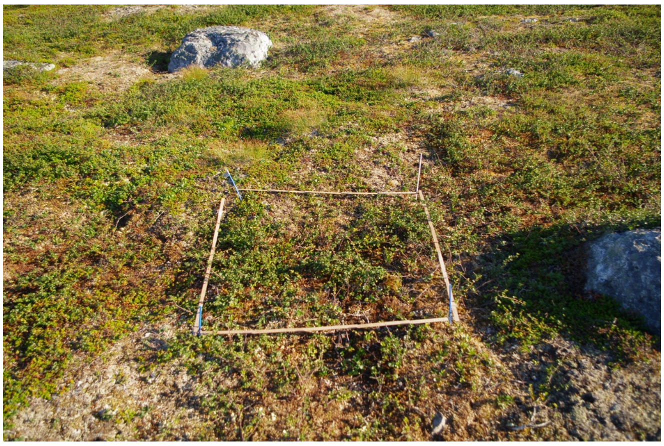 VJ-12
		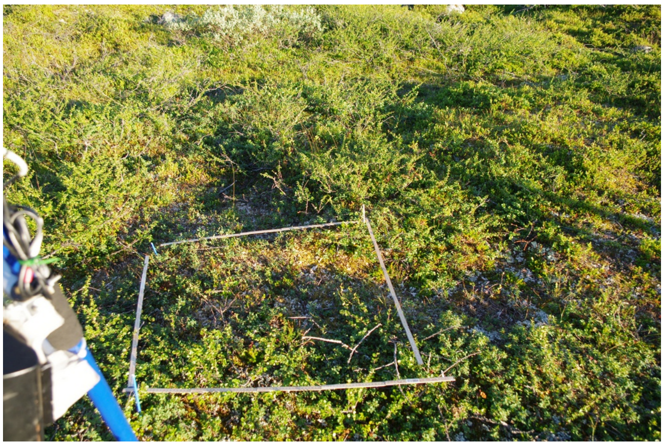 VJ-20

Willow	Willow (*Salix spp.)* erect shrub communities standing between 0.5–2 m height. Typically occupying wind sheltered depressions, as well as wetland edges and stream banks. Can stand on intermediate to seasonally waterlogged ground.	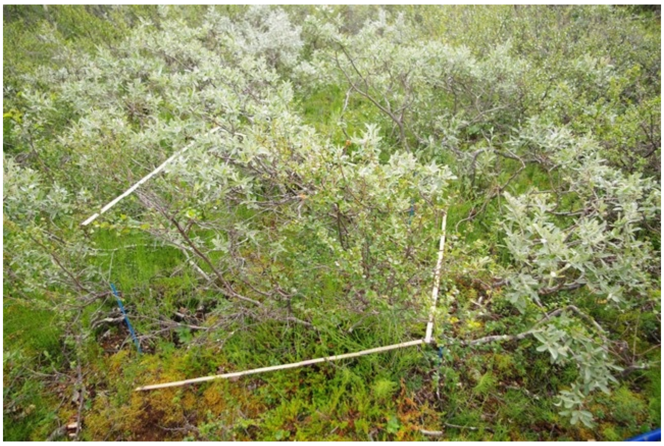 NF-31
		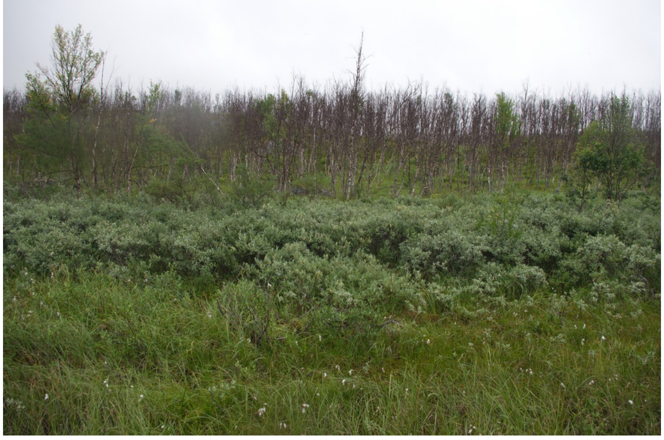 VJ-31
		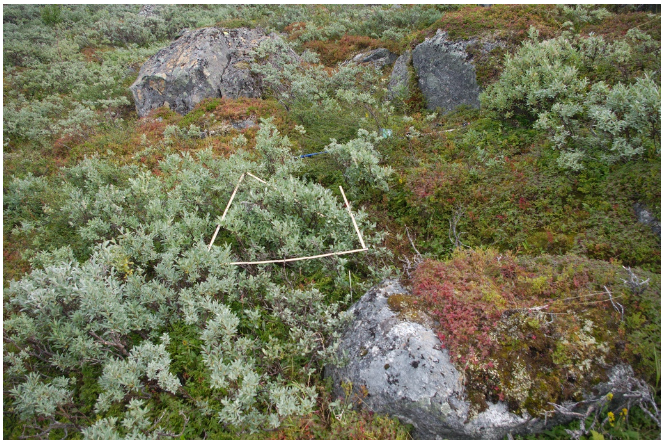 VJ-09
Snowbed	Areas with persistent snowbeds long into the season. Often marked by moss and lichen communities and snowbed species like *Salix herbacea*.	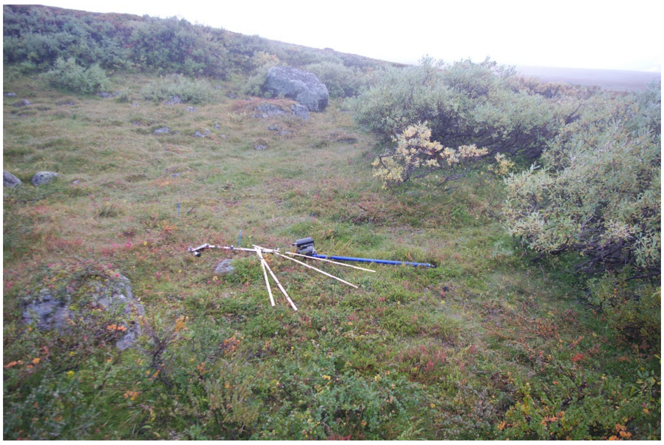 NT-12

		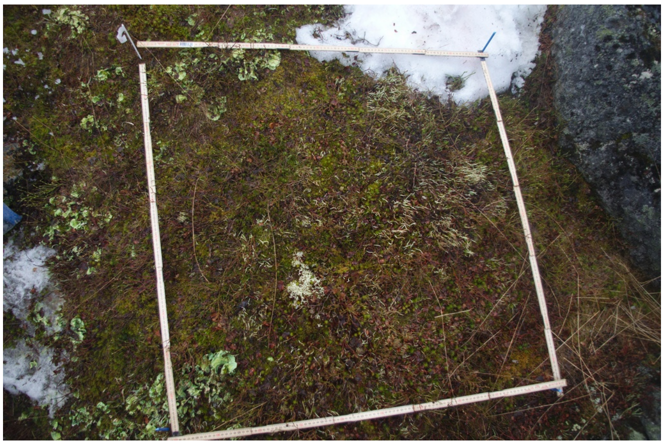 NT-27
Semi-wetland	Semi-wetland or moist tundra, seasonally flooded or areas with frequent ponds caused by patterned ground.	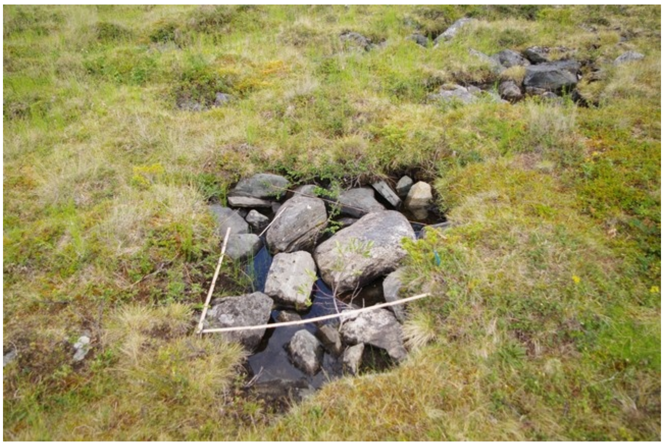 NF-11
		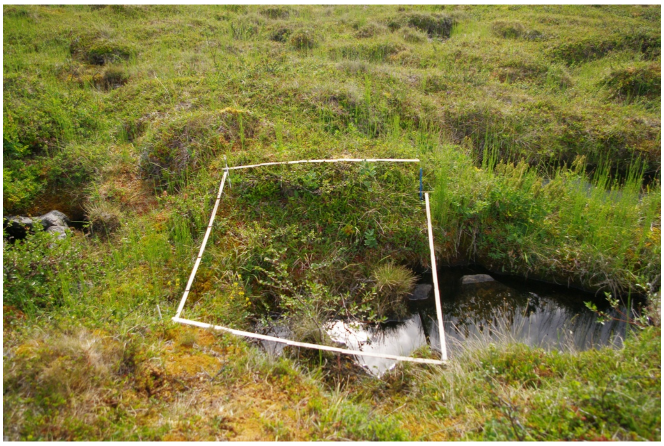 NF-24
		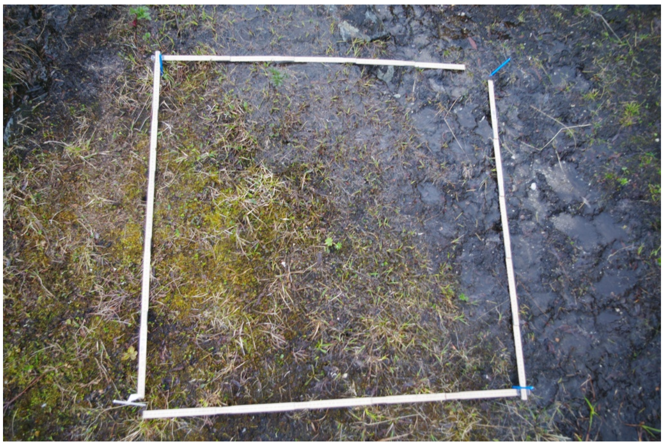 KJ-08

Graminoid meadow	Graminoid dominated meadows that are seasonally flooded.	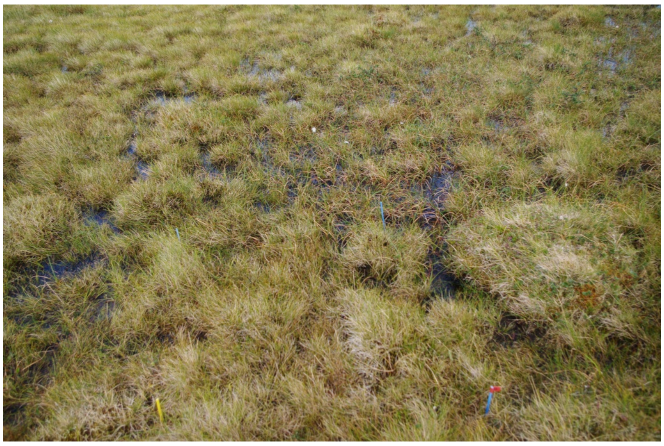<br>NT-31
		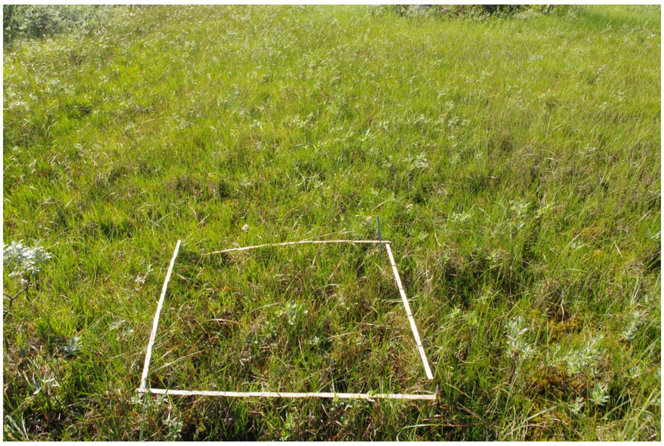<br>VJ-18
Wetland	Typically *Sphagnum ssp.* dominated areas or longtime inundated areas but higher water table than graminoid dominated meadows. Presumably with deep peat layers.	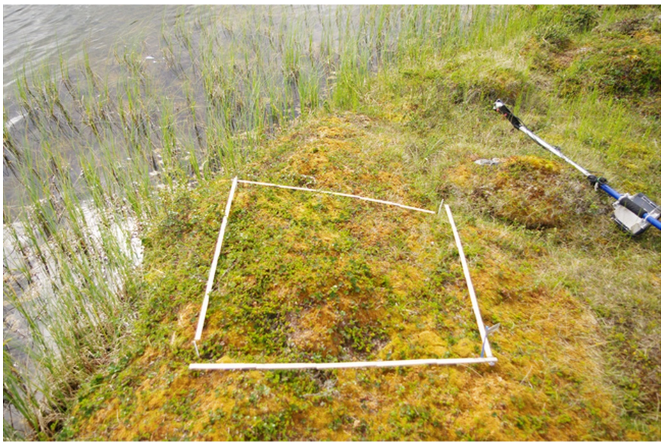<br>NF-4
		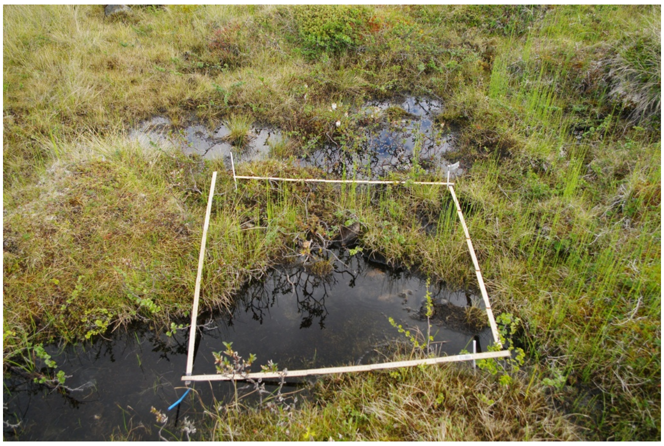<br>NF-02

Birch Forest	Mountain Birch forest (*Betula pubescens ssp. czerepanovii)* includes both wet and dry ground conditions.	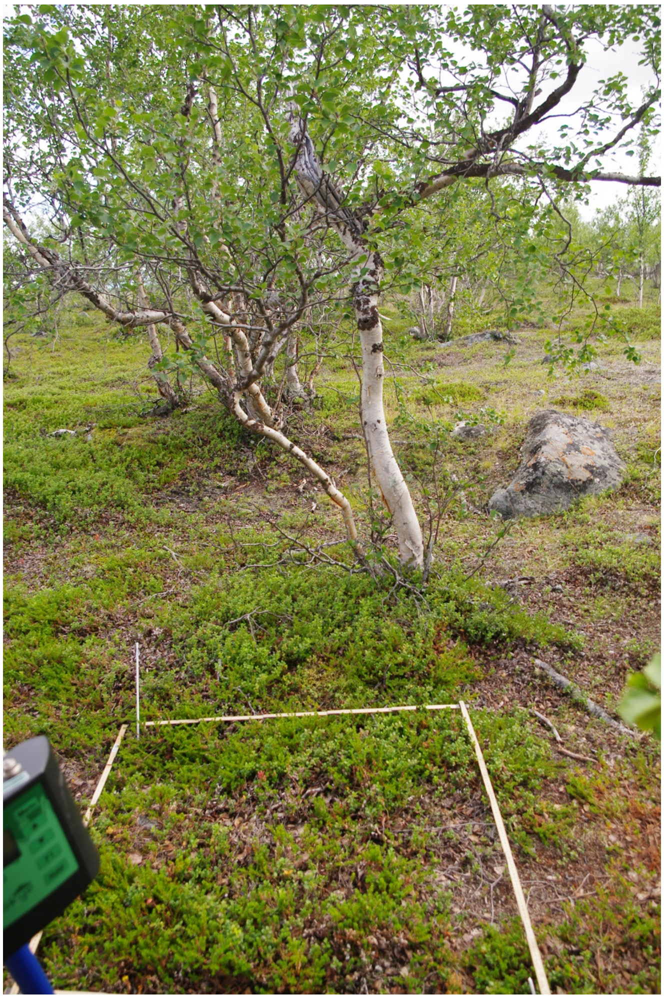 NF-17
		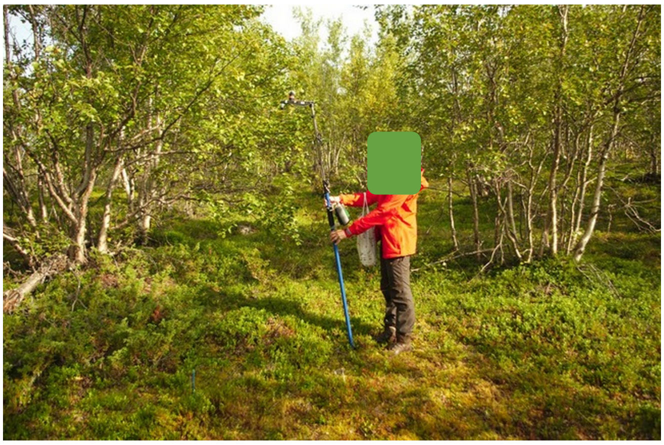 NF-20

		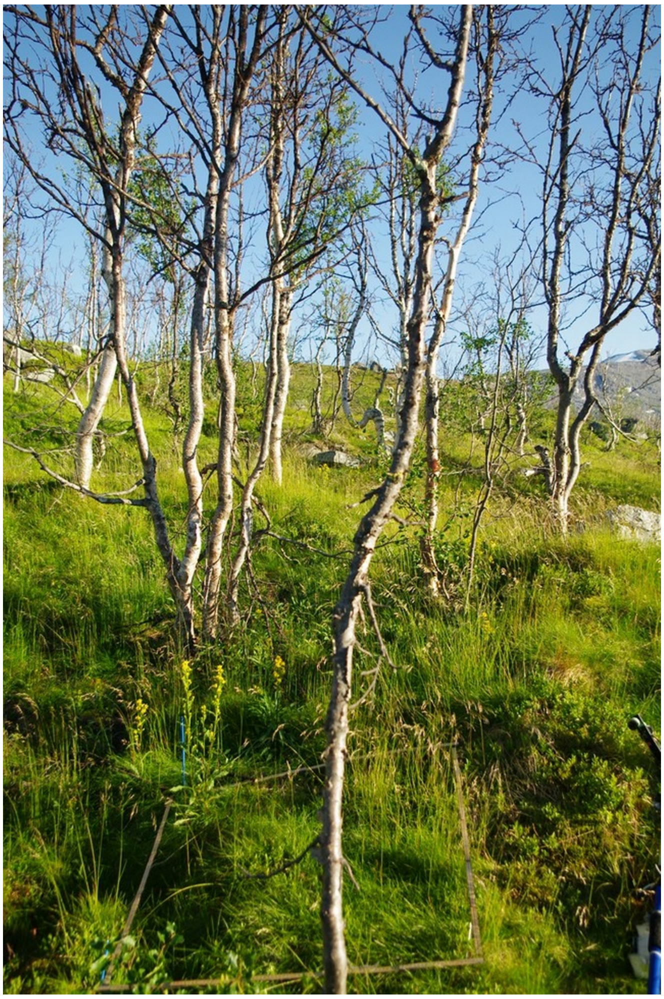<br>VJ-11

Supplementary Discussion

Land cover classification accuracy assessment

The out-of-bag (OOB) error rates of the original random forest model ranged between 0.3% to 6% for the individual study areas (Table S1). The Overall Accuracy and Kappa Coefficient were derived from 33 randomly distributed ground control points within each study area (see Siewert & Olofsson[1]) and ranged between 0.61 and 0.70 for the Overall Accuracy, from 0.50 to 0.65 for the Kappa Coefficient. These values indicate moderate to good agreement and are within reach of other Arctic land cover classifications[2–5]. Given the accuracy assessment, the very high-resolution of UAV input data and judgment from our field knowledge, we are overall confident that the land cover classifications correctly identify major habitat types in these study areas.

Table S1| Accuracy assessment values of the land cover classifications.

Study area	OOB estimate of error rate	Overall Accuracy	Kappa
NF	3.22%	0.67	0.60
NT	5.67%	0.58	0.50
VJ	0.97%	0.61	0.55
KJ	0.32%	0.70	0.65

References

1. Siewert, M. B. & Olofsson, J. Scale-dependency of Arctic ecosystem properties revealed by UAV. *Environ. Res. Lett.* **15**, 094030 (2020).
2. Siewert, M. B. High-resolution digital mapping of soil organic carbon in permafrost terrain using machine learning: a case study in a sub-Arctic peatland environment. *Biogeosciences* **15**, 1663–1682 (2018).
3. Siewert, M. B., Hugelius, G., Heim, B. & Faucherre, S. Landscape controls and vertical variability of soil organic carbon storage in permafrost-affected soils of the Lena River Delta. *CATENA* **147**, 725–741 (2016).
4. Siewert, M. B. *et al.* Comparing carbon storage of Siberian tundra and taiga permafrost ecosystems at very high spatial resolution: Ecosystem carbon in taiga and tundra. *Journal of Geophysical Research: Biogeosciences* **120**, 1973–1994 (2015).
5. Virtanen, T. & Ek, M. The fragmented nature of tundra landscape. *Int. J. of Appl.Earth Obs. a. Geoinfo.* **27, Part A**, 4–12 (2014).
